# Metagenomic Analysis from the Interior of a Speleothem in Tjuv-Ante's Cave, Northern Sweden

**DOI:** 10.1371/journal.pone.0151577

**Published:** 2016-03-17

**Authors:** Marie Lisandra Zepeda Mendoza, Johannes Lundberg, Magnus Ivarsson, Paula Campos, Johan A. A. Nylander, Therese Sallstedt, Love Dalen

**Affiliations:** 1 Centre for GeoGenetics, University of Copenhagen, Natural History Museum of Denmark, Copenhagen, Denmark; 2 Department of Botany, Swedish Museum of Natural History, Stockholm, Sweden; 3 Department of Palaeobiology and the Nordic Center for Earth Evolution (NordCEE), Swedish Museum of Natural History, Stockholm, Sweden; 4 Department of Bioinformatics and Genetics, Swedish Museum of Natural History, Stockholm, Sweden; Universiteit Utrecht, NETHERLANDS

## Abstract

Speleothems are secondary mineral deposits normally formed by water supersaturated with calcium carbonate percolating into underground caves, and are often associated with low-nutrient and mostly non-phototrophic conditions. Tjuv-Ante’s cave is a shallow-depth cave formed by the action of waves, with granite and dolerite as major components, and opal-A and calcite as part of the speleothems, making it a rare kind of cave. We generated two DNA shotgun sequencing metagenomic datasets from the interior of a speleothem from Tjuv-Ante’s cave representing areas of old and relatively recent speleothem formation. We used these datasets to perform i) an evaluation of the use of these speleothems as past biodiversity archives, ii) functional and taxonomic profiling of the speleothem’s different formation periods, and iii) taxonomic comparison of the metagenomic results to previous microscopic analyses from a nearby speleothem of the same cave. Our analyses confirm the abundance of *Actinobacteria* and fungi as previously reported by microscopic analyses on this cave, however we also discovered a larger biodiversity. Interestingly, we identified photosynthetic genes, as well as genes related to iron and sulphur metabolism, suggesting the presence of chemoautotrophs. Furthermore, we identified taxa and functions related to biomineralization. However, we could not confidently establish the use of this type of speleothems as biological paleoarchives due to the potential leaching from the outside of the cave and the DNA damage that we propose has been caused by the fungal chemical etching.

## Introduction

Speleothems are secondary mineral deposits, most of them form when water supersaturated with calcium carbonate percolates down to caves and precipitates carbonate minerals, normally in the form of calcite [1]. Speleothems are an interesting environment to study given the composition and the chemical process of its formation. These aspects have an impact in its microbiome, which should be adapted to the speleothem’s mostly non-phototropic and low-nutrient characteristics, as well being capable of performing calcite precipitation (i.e. biomineralization). And given the long time required for its formation, the study of the DNA in different formation periods in the speleothem could provide information on species (e.g. bacteria or higher-level eukaryotes living above the cave) present in the past, at the time of its formation. However, no study has been performed on the interior of them and compared different vertical samples representing different formation points.

A few studies, most of them based on microscopy and DNA metabarcoding, have been done to analyze the taxonomical and functional characteristics of the microbiome from the surface of speleothems [2–6]. Bacterial profiling from the surface of speleothems from a limestone cave in Arizona showed that DNA from *Actinobacteria* and *Proteobacteria* dominated the samples [4]. And a functional study on the speleothem surface microbiome from a carbonate cave suggested that it is adapted to low-nutrient conditions through the use of nitrogen as the main energy-production strategy with contributions from archaea and bacteria, as well as through the use of CO_2_-fixation pathways [6]. Given that carbon isotope fractionation rates vary with different microbial CO_2_-fixation pathways, this highlights the importance of understanding the microbial contributions to speleothem isotopic signatures [7] when speleothems are used as climate archives through the use of carbon isotopes. However, these studies do not explore the use of speleothems as past biodiversity archives, functional and taxonomic profiling of the speleothem’s different formation periods. Also, so far there has not been a direct comparison of microscopic to DNA shotgun metagenomic datasets from speleothems of the same cave. In this study we aim at exploring these three aspects using a speleothem from Tjuv-Ante’s cave.

Tjuv-Ante’s cave is situated at an elevation of 90 meters above sea level in Storrisberget’s Nature Reserve on the north-eastern Swedish coast at N 63° 35.6’, E 19° 22.8’. It belongs to a rare kind of caves, given that it was formed by wave abrasion in a dolerite dyke intruded in granite gneiss and so the walls are of granite and the ceiling of dolerite [8,9]. Most caves studied so far have a more stable microclimate with temperatures ranging from 13 to 15°C, while Tjuv-Ante’s cave, due to its shallow depth, has a much more variable microclimate closely tracking the temperature variation of the surface above the cave, ranging from -10°C to 15°C [10].

The process of speleothem formation in this cave is of particular interest because the main speleothem forming mineral is calcite, which is not reported as a major speleothem forming mineral in granite caves [5]. A previous study combining Environmental Scanning Electron Microscope (ESEM) and fluorescent microscopy on this cave has shown an abundance of fungi and *Actinobacteria* that together play important roles in the speleothem life cycle through a constructive-destructive interplay [5]. *Actinobacteria* are commonly identified in cave biofilms [11], speleothems, and cave soils from various locations, and can induce mineral precipitation [12,13], while fungi are mostly destructive agents [14] due to chemical etching and physical breakdown of the mineral substrate.

It has been suggested that the speleothem microbiome from Tjuv-Ante’s cave consists of heterotrophs probably living of organic matter transported from the surface by percolating fluids [5]. However, it is not known if there are any chemoautotrophs in the dolerite that can act as a carbon source for the heterotrophic communities on the cave walls. In Tjuv-Ante’s cave, microbial biofilms are absent from the granite walls, but present all over the dolerite ceiling and are also incorporated within the dark layers of the speleothem, which are rich in organic compounds and opal-A (an amorphous, hydrated silica mineraloid). The speleothem is thought to grow seasonally, with the biofilms extensively growing during spring and summer and then being mineralized and overgrown by calcite during autumn and winter, thus the biofilms may play a role in the speleothem formation [5].

Given the slow deposition of mineral in the speleothem, this environment provides an interesting substrate for its evaluation as paleo biodiversity archives. Deep ice cores, permafrost, and sediments analyzed with genetic techniques have been used to identify plant and animal DNA that has been preserved there since the Holocene and Pleistocene. This has allowed the reconstruction of paleo fauna and paleo vegetation even in the absence of obvious macrofossils [15,16]. Importantly, these studies have been possible not only from cold environments, but also from temperate cave sediments including dry silty sediments from a subalpine cave in New Zealand [15].

These studies are possible due to the ability of the environmental DNA to bind to soil mineral particles through ionic interactions between the negatively charged phosphate groups of DNA and positively charged surface groups [17]. If the surface is positively charged, the low pH functional groups of the nucleic acids may be the ones playing part in the adsorption [18]. The adsorption strength of DNA to different mineral surfaces has been tested, including calcite and silica [19,20]. It is also believed that DNA extraction of microbial communities in consolidated sediments poses extraction difficulties due to the DNA being bound to the silica sediment, which is known to effectively bind DNA at neutral pH [19,21,22].

**Fig 1:**
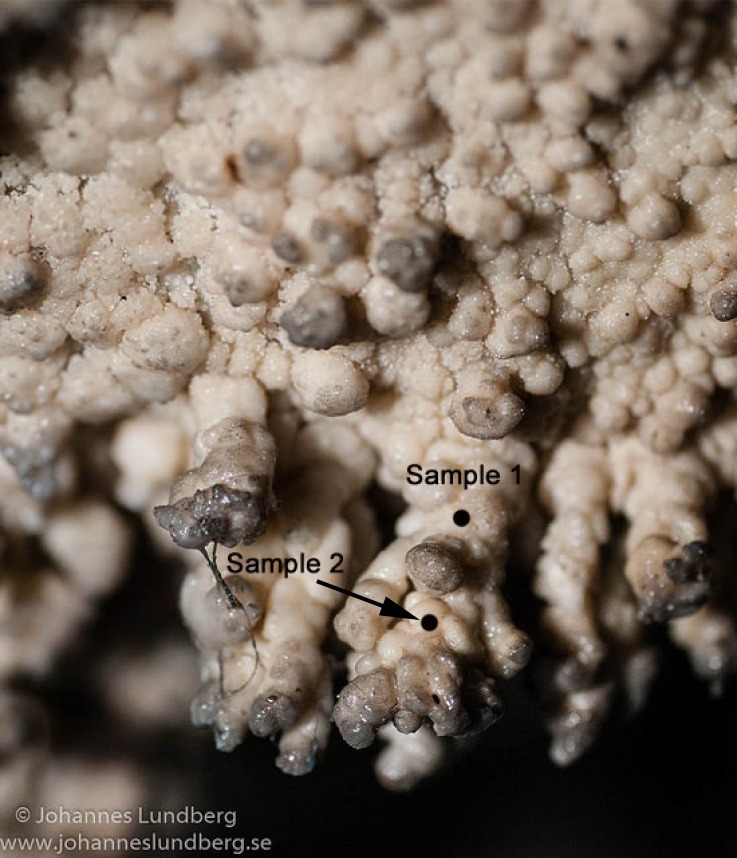
Tjuv-Ante’s cave sampled speleothem type. Sample 1 was taken from the basal part of the speleothem, from a relatively old formation. Sample 2 was taken from a higher up location in the speleothem and comes from a relatively recent formation.

In order to characterize the microbiome of a speleothem from Tjuv-Ante’s cave by performing a deep functional and taxonomic profiling, we analyzed samples drilled from two different locations from the dark layers of the interior of the speleothem. A speleothem of similar size and close proximity to the sampled one has been radiocarbon dated to 1259 BP [5], serving as a proxy for the age of the sampled speleothem. One sample (from here on called sample 1) was taken from the interior of a relatively basal part of the speleothem, and the other (from here on called sample 2) was taken from the interior of a location closer to the tip of the speleothem (Fig 1). Thus, the samples represent different formation dates, an older and a more recent one, respectively. In contrast to previous DNA studies, we focused not only on identifying bacteria and archaea, but also fungi, algae, virus, protozoa, as well as non-microbial species given our use of shotgun DNA sequencing metagenomic approach. Our analyses confirm the previous microscopy findings and suggest the presence of chemoautotrophs; furthermore, we identified taxa and functions related to biomineralization. However, the use of speleothems as biological paleoarchives could not be confidently established.

## Materials and Methods

### Sample collection

We are very thankful to the Länsstyrelsen Västerbotten, which granted us a sampling permit for Tjuv-Ante’s cave (case number 521-6864-2010). The speleothem samples were collected as described in [5]. Briefly, sampling was done using a Multitool drill (Dremel) to obtain ca. 30 mg of calcite powder from two places of the interior of the speleothem. One sample (sample 1) comes from a basal part of the speleothem, and the other (sample 2) comes from closer to the tip of the speleothem. Sterile tools were used and the samples were wrapped in aluminum foil after sampling. Moreover, the samples were only handled with stainless steel forceps and not touched by ungloved hands. Afterwards, they were stored on ice and transferred to a -20°C freezer where they were stored until analysis. For more information on the cave see S1 File.

### DNA extraction and sequencing

DNA from the calcite powder was extracted following the approach outlined in [23]. Briefly, DNA was extracted using a silica-based method. Roughly 15–50 mg calcite powder from each sample was incubated under motion overnight at 55°C in 715 μl extraction buffer (0.45M EDTA, 0.1M urea, 150 μg proteinase K) [24]. Following this, the samples were centrifuged at 2,300 rpm for 5 minutes and the supernatants were collected and concentrated to a volume of 20–100 μl using 30K MWCO Vivaspin filters (Sartorius). The concentrated supernatants were subsequently mixed with 5X PB buffer (Qiagen), purified on Qiaquick silica spin columns using PE buffer (Qiagen). Unfortunately, the DNA concentration was too low to visualize the size distribution on a gel. Afterwards, the supernatants were eluted twice using 2x50μl EB buffer (Qiagen). Aliquots from the DNA extracts were subsequently converted into Illumina libraries using a NEBNext^®^ DNA Library Prep Master Mix Set 2 (New England Biolabs, E6070) following the manufacturer’s instructions with the following modifications. The extracts were not nebulized; reaction volumes were cut down from the manufacturer’s protocol by a quarter in the end-repair step and by half in the ligation and fill-in steps. After the end-repair and ligation incubations, the reaction was purified through MinElute spin columns and eluted in 15 μl and 21 μl volumes, respectively, after a 5-minute incubation at 37°C with Qiagen EB. Ligation reaction was performed for 25 minutes at 20°C using Illumina-specific adapters specified in [25]. A fill-in reaction was performed for 20 minutes at 65°C. Libraries were amplified in two rounds.

The purified libraries were amplified as follows: 5 μl DNA library, 1X High Fidelity PCR buffer, 2 mM MgSO4, 200 μM dNTPs each (Invitrogen, Carlsbad, CA), 200 nM Illumina Multiplexing PCR primer inPE1.0 (5’AATGATACGG CGACCACCGA GATCTACACT CTTTCCCTAC ACGACGCTCT TCCGATCT), 4 nM Illumina Multiplexing PCR primer inPE2.0 (5’GTGACTGGAG TTCAGACGTG TGCTCTTCCG ATCT), 200 nM Illumina Index PCR primer (5’CAAGCAGAAG ACGGCATACG AGATNNNNNN GTGACTGGAG TTC, where N’s correspond to a 6 nucleotide index tag), 1 U of Platinum Taq DNA Polymerase (High Fidelity) (Invitrogen, Carlsbad, CA) and water to 50 μl. Cycling conditions were: initial denaturing at 94°C for 4 minutes, 18 cycles of 94°C for 30 seconds, 59°C for 30 seconds, 68°C for 40 seconds, and a final extension at 72°C for 7 minutes. PCR products were purified through MinElute spin columns and eluted in 10 μl of Qiagen Buffer EB, following a 10-minute incubation at 37°C. A second round of PCR (two parallel reactions for each library) was set up as follows: 5 μl of purified product from the first PCR round, 1X High Fidelity PCR buffer, 2 mM MgSO4, 200 μM dNTPs each, 500 nM Illumina Multiplexing PCR primer 1.0, 10 nM Illumina Multiplexing PCR primer 2.0, 500 nM Illumina Index PCR primer, 1 U of Platinum Taq DNA Polymerase (High Fidelity), and water to 50 μl. Cycling conditions included an initial denaturing at 94°C for 4 minutes, 12 cycles of: 94°C for 30 seconds, 59°C for 30 seconds, 68°C for 40 seconds, and a final extension at 72°C for 7 minutes. All libraries were run on a 2% agarose gel and size selected 150–300 bp. All products were purified with Qiagen QIAquick gel extraction kit (Qiagen, Valencia, California). Samples were pooled equimolarly and sequenced on one lane of llumina HiSeq 2000 (100 cycles, single-end read mode) at the Danish National High-Throughput DNA Sequencing Centre.

### Sequence length distribution

We used MGmapper v1.07 [26] to map the datasets against the next databases: human, bacteria, virus, fungi, protozoa, invertebrates, toxin, mammalian vertebrates, other vertebrates, and common meat (pig, cow, chicken, and sheep). These databases consist of whole genome entries obtained from NCBI. Subsequently, we looked at the reads length distribution from the results in order to differentiate endogenous DNA from contaminant DNA [27]. To this end, we plotted with R v2.15.2 the density distribution of the length of the reads mapping uniquely to the bacteria, fungi, and protozoa databases. To test for multimodality on the distributions we used the CRAN package diptest [28] that implements the Hartigan's dip test statistic (D). The dip test measures multimodality in a sample by the maximum difference, over all sample points, between the empirical distribution and the unimodal distribution function that minimizes that maximum difference. The p value is calculated by comparing the D obtained with those for repeated samples of the same size from a uniform distribution. Specifically, we used the “dip.test” function with the parameter B set to 2000 and the p value to be calculated via linear interpolation. We then compared the taxa identified by uniquely mapping long (>90 nts) and short (<90 nts) reads and mined those microbes identified only by long reads. Furthermore, in order to test whether shorter reads originate from ancient specimens trapped in the speleothem, reads mapping to the Bacteria database with a length up to a certain threshold selected based on the hit length distribution were classified as short (<50 nts) or long (>70 nts) and the ancient pattern was tested with MapDamage v2 [29]. MapDamage approximates a bayesian estimation of damage parameters. The examined damage patterns are i) the probability of terminating in overhang, ii) the cytosine deamination probability in double strand context, iii) the cytosine deamination probability in single strand context, and iv) the mean difference rate between the reference and the sequenced sample not due to DNA damage. When counting misincorporations, the impact of sequencing errors is taken into account by setting a quality threshold.

### Taxonomic and functional profiling

Using the taxonomic hits obtained with MGmapper, we generated taxonomic profiles from phylum to the species level using the biom format v1.1.1 [30] and Qiime [31]. We also analyzed the dataset with MG-RAST v3 [32], from where they are publicly available under the ids 4571211.3 for sample 1, and 4571212.3 for sample 2. In MG-RAST the optional dereplication step was used, in which redundant technical replicate sequences are removed. One copy of each 50 bp identical bin was retained. Briefly, the functional profiling in MG-RAST works by first predicting coding regions within the sequences using FragGeneScan [33], an *ab initio* prokaryotic gene-calling algorithm. After this prediction, sequences are clustered with 90% identity. The functional profiling was performed using the KEGG and Subsystems annotations. We used a maximum e-value of 1e-5, a minimum identity of 60% and 75%, and a minimum alignment length of 15 measured in amino acids for protein and base pairs for RNA databases. Heat maps were obtained with Ward clustering with Bray-Curtis distance metric using normalized counts.

Briefly, the taxonomic profiling in MG-RAST for each sample is performed by first pre-screening the sequences using QIIME-UCLUST [31,34] for at least 70% identity to ribosomal sequences from the following RNA databases: Greengenes [35], Silva LSU and SSU [36], and RDP [37]. Afterwards, sequences are clustered *de novo* at 97% identity using QIIME-UCLUST. We also perform taxonomic profiling using the databases M5NR, KEGG, and RefSeq. For the taxonomic comparison of both samples, the data was compared using a maximum e-value of 1e-5, a minimum identity of 75% and 60%, and a minimum alignment length of 15 measured in amino acids. Comparison of the two samples was made with Ward clustering with Bray-Curtis distance metric, grouped by phylum and class using normalized values. The comparison was also visualized through a tree with the lowest common ancestor. The data was compared using a maximum e-value of 1e-5, a minimum identity of 75%, and a minimum alignment length of 15 measured in amino acids from the M5NR database. Leaf weights were displayed as stacked bar chart maximum level order and colored by phylum.

Furthermore, we generated a *de novo* assembly of the two metagenomes using Ray Meta v2.3.2-devel [38]. Subsequently we predicted genes using prodigal v2.6 [39], and then blasted the predicted genes against the nt database and analyzed the taxonomy of the results with MEGAN [40]. Furthermore, as a second means of exploring the origin of the short and long reads, we mapped the reads cleaned with MOCAT [41] to the assembled scaffolds and examined the lengths of the hits. Based on the read length distribution we classified as short reads those less than 50 nts long, and as long those longer than 70 nts. Afterwards the ancient signal was tested on the two groups with MapDamage v2 [29].

In order to test for significantly abundant bacterial taxa and functions in both samples as well as differentially abundant bacterial taxa between the samples, we used the species level identifications from MGmapper against the bacteria database using only the unique mapping reads and the level 3 functions annotated from KEGG. We obtained the Bonferroni corrected p values of the counts normalized by percentage under a Poisson distribution.

## Results

### Sequence length distribution

**Fig 2:**
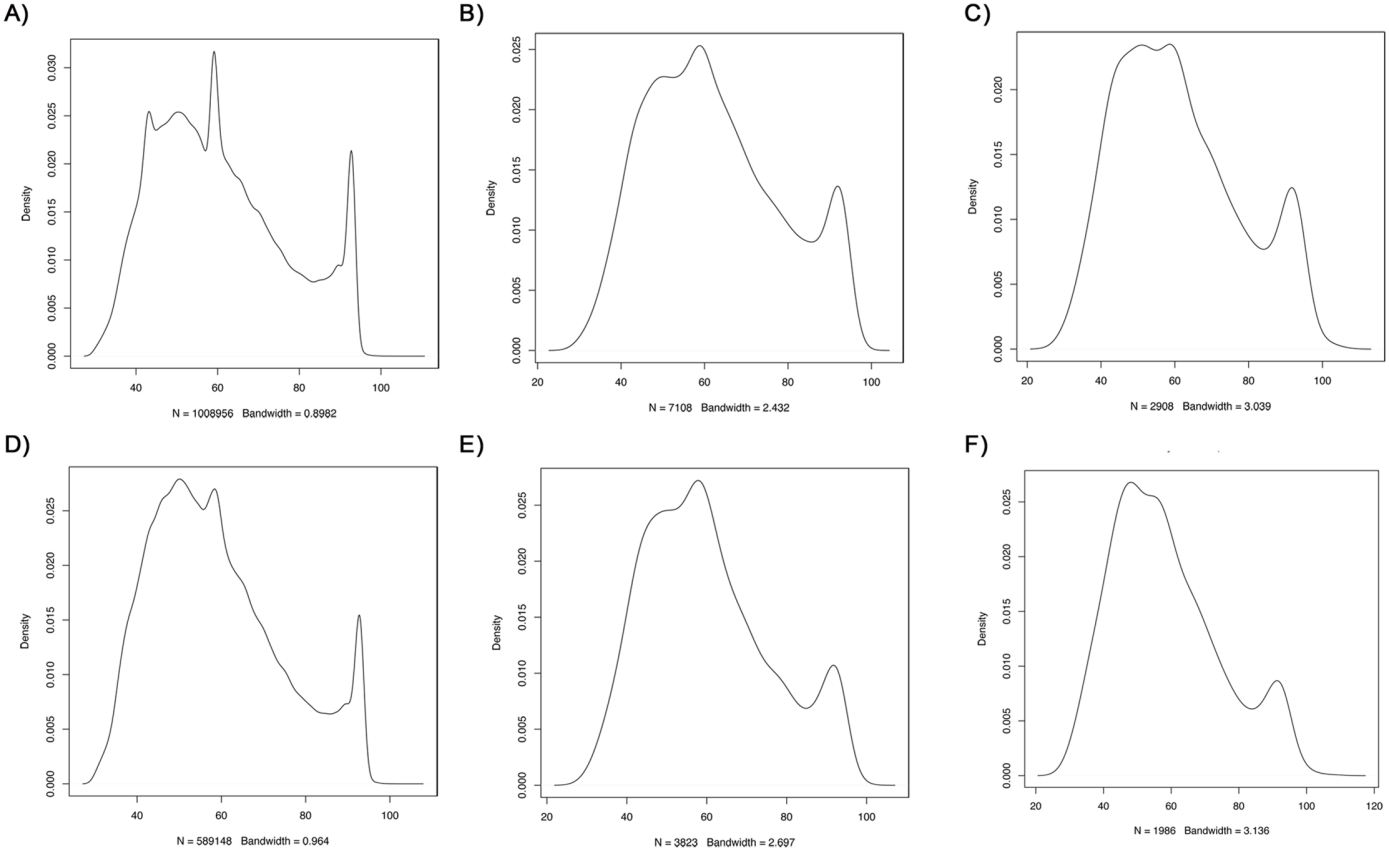
Density plot of the DNA fragments length distribution from the two sampled metagenomes mapping uniquely to different genome databases. (A) Bacteria from sample 1, p-value < 2.2e-16 (B) Fungi from sample 1, p-value < 2.2e-16 (C) Protozoa from sample 1, p-value < 2.2e-16 (D) Bacteria from sample 2, p-value < 2.2e-16 (E) Fungi from sample 2, p-value < 2.2e-16 (F) Protozoa from sample 2, p-value = 2.7e-05.

**Fig 3:**
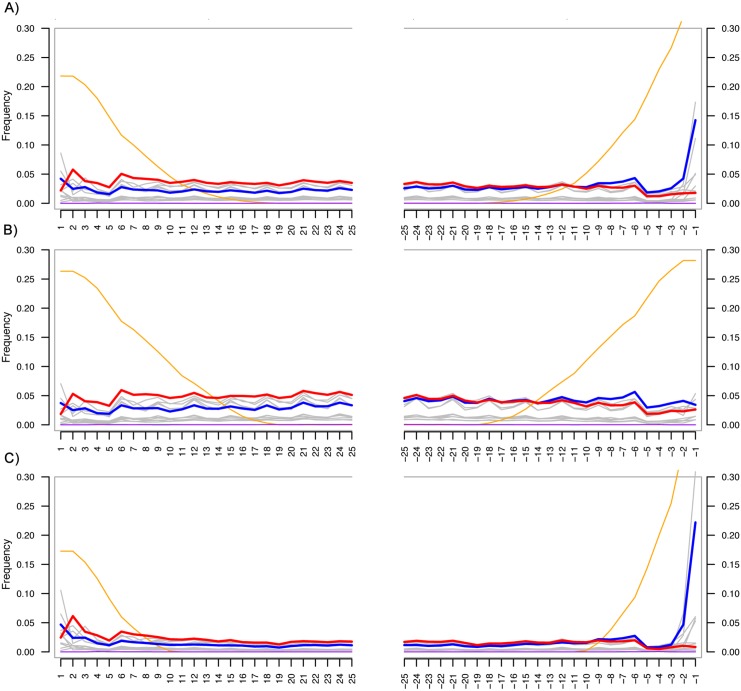
Damage pattern. Damage pattern on the reads from sample 1 uniquely mapping to the bacteria database in MGmapper from (A) all the mapping reads, (B) the subset of long reads, (C) the subset of short reads. C to T damage is depicted in red color, and G to A is depicted in blue color. Grey lines represent other nucleotides derived from other types of DNA damage. The orange line represents soft-clipped bases, those that are not aligned to the reference.

We tried to differentiate DNA derived from contamination or DNA leaching from the surface above the cave from actual fossil DNA from microorganisms trapped within the speleothem during the growth and/or recent DNA from microorganisms native to the interior of the speleothem. To this end, we first investigated the distribution of the length of the reads mapping uniquely to bacteria, fungi and protozoa. In both samples we found a bimodal distribution on the tested datasets of bacteria, fungi, and protozoa from both samples, with the majority of the reads falling in the short length range of the distribution (Fig 2). As a second method to test for the ability to differentiate ancient from modern DNA, we analyzed the damage pattern of the reads mapped to the bacteria database (Fig 3, S1 Fig) and the *de novo* assembled contigs (S2 and S3 Figs), separating the hits of the long and the short reads. We identified damage at both ends of the reads characteristic of ancient DNA (aDNA) (Fig 3, S1–S3 Figs), however we also observe other types of substitutions. Thus, the damage characteristic of aDNA cannot be uniquely distinguished in the reads since also other types of damage are present.

### Taxonomic profiling

In MG-RAST, from the initial 44,846,754 sequences from sample 1 and the 32,491,609 sequences from the dataset from the sample 2, 66.9% and 71.9% of the datasets passed the quality control with mean sequence length of 89 ± 9 bp, respectively (S1 Table). From the sequences that passed the quality control steps from sample 1, a total of 1,004,935 sequences (2.2%) contain ribosomal RNA genes. The cleaned dataset from sample 2 contains 587,443 sequences (1.8%) from rRNA genes (S2 Table). Using the ribosomal genes the α-diversity of sample 1 is 381.111 species and 533.045 species for the second sample, and 301.02 and 421.62, respectively, when using the RefSeq database. The saturation curve from both samples does not reach a plateau, with both samples closely together in the exponential phase (S4 Fig). MGmapper identified a total of 1,008,956 and 589,148 reads mapping to bacteria from samples 1 and 2, respectively, and a total of 10,265 in sample 1 and 5,989 in sample 2 mapping to the virus, fungi, and protozoa databases (S3 Table).

We *de novo* assembled a total of 6,645 contigs larger than 500 nts in sample 1, and 723 from sample 2. The N50 is 947 nts for sample 1, and 742 for sample 2. The largest contig from sample 1 is 11,043 nts and 5,262 nts from sample 2. From the *de novo* assembly we predicted 80,393 genes from sample 1 and 66,557 from sample 2.

Results from MGmapper could identify a total of 2,908 reads uniquely mapping to protozoa species in sample 1, and 1,986 in sample 2. The most abundant protozoa in both samples were *Physarum polycephalum* with 705, and 467 uniquely mapping reads in sample 1 and sample 2, respectively. The following most abundant protozoa on sample 1 are the alga *Nannochloropsis* and the plant pathogen *Pythium* with 246 and 228, respectively. For sample 2 the following most abundant protozoan species are *Pythium* and the alga *Aureococcus* with 193 and 172, respectively. Virus identified by MGmapper total only 249 unique mapping reads from sample 1 and 180 on the second sample. Among them, the most abundant phage was *Streptococcus* phage with 32 and 23 unique mapping reads from sample 1 and 2, respectively. Those identified by MG-RAST at the phylum level only include *Herpesvirales* and *Caudovirales*.

When taking into account only the uniquely mapping long reads from the MGmapper results, a similar number of bacteria, fungi and protozoa database entries were identified on both samples. Sample 1 contained 884 bacteria, 172 protozoa, and 117 fungi identified by long uniquely mapping reads, and sample 2 contained 834, 100, and 68 respectively. When removing the hits with both long and short reads and keeping those identified by only long reads, the number of hits to the databases was largely reduced. Sample 1 contains 28, 94, and 29 hits to the databases of bacteria, protozoa, and fungi databases, respectively. Sample 2 contains 16, 60, and 24, respectively.

**Fig 4:**
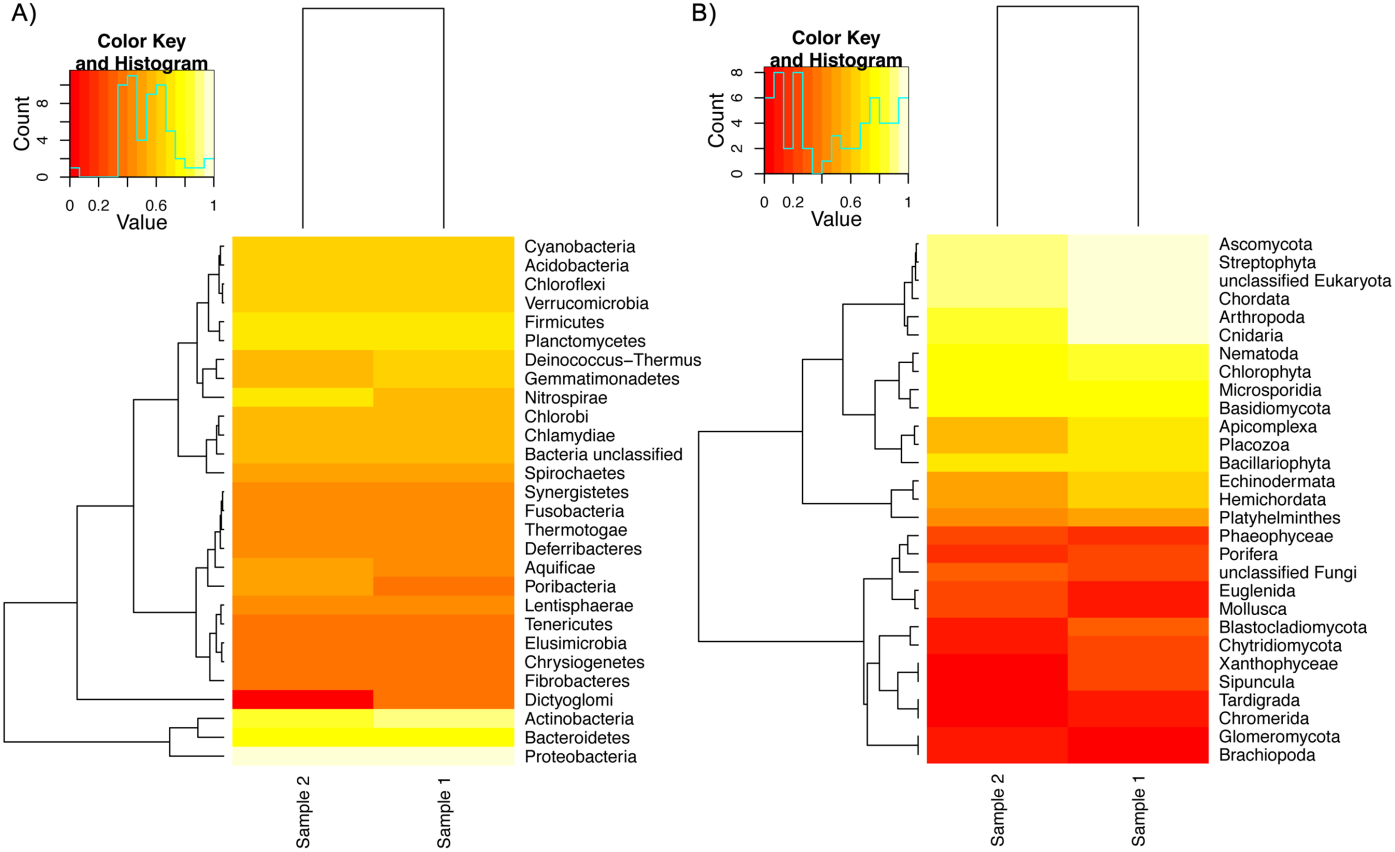
Phylum taxonomic level heat map derived from mapping against the RefSeq database. (A) Bacteria phylum. (B) Eukaryota phylum. The most abundant bacterial phyla are *Proteobacteria*, *Actinobacteria*, and *Bacteroidetes*. *Cyanobacteria* are also present at high abundance. Different phyla of fungi are also present at high abundance, however we also identify higher-level eukaryotes like insects, worms, and marine species, at both large and low abundances.

**Fig 5:**
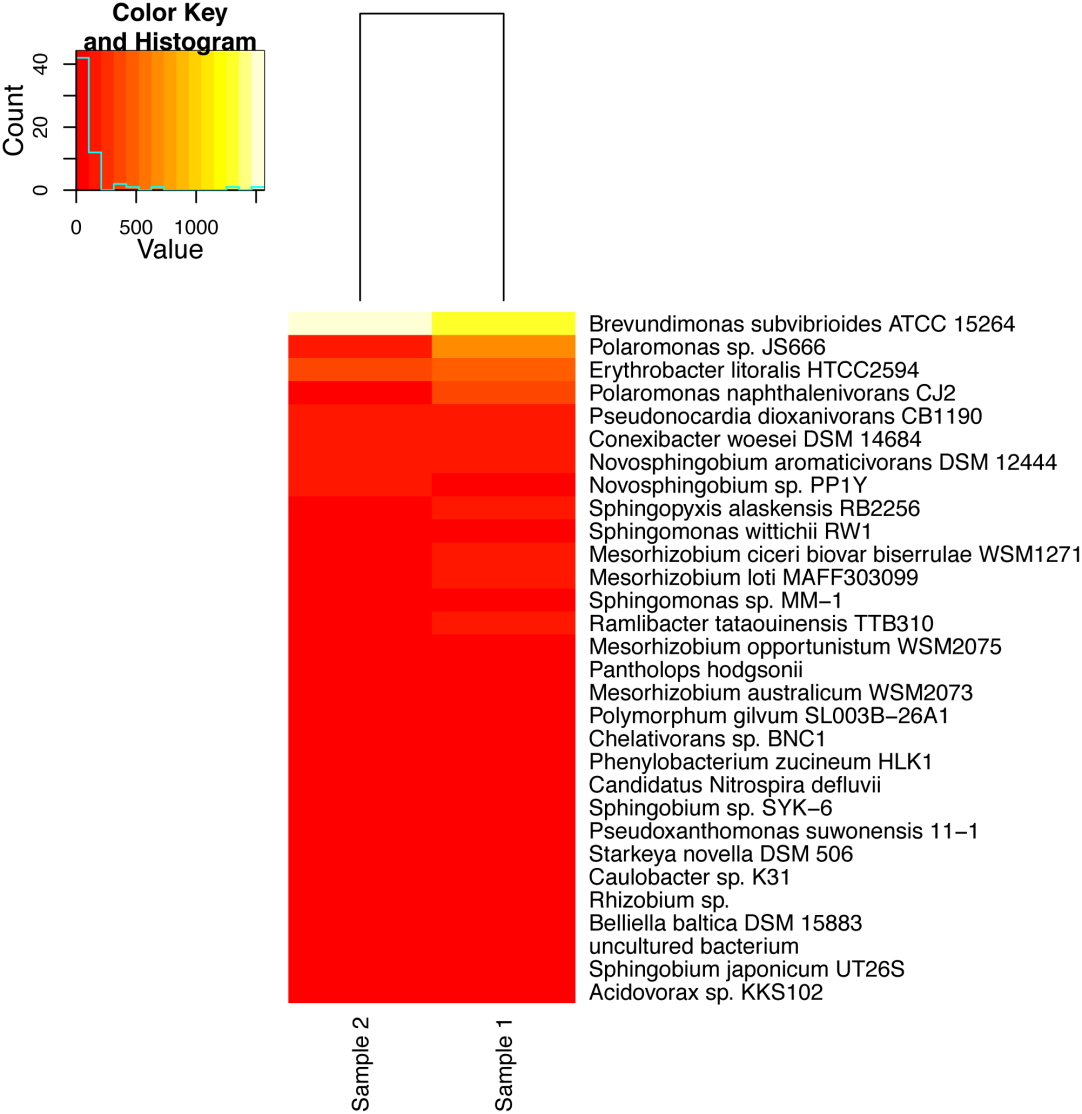
Top 30 most abundant species identified from the *de novo* predicted genes.

In general both samples share very similar taxonomic composition at the phylum (Fig 4, S5 Fig) and order levels (S6 Fig). In both samples, the most abundant taxa are the class *Alphaproteobacteria* (629,932 sequences in sample 1, and 268,869 in sample 2), followed by the class *Actinobacteria* (200,786 sequences for sample 1 and 120,571 for sample 2) (S7 Fig, S2 File). A species level comparison using the MEGAN identifications from our *de novo* assembled and predicted genes, highlights *Brevundimonas subvibrioides*, two *Polaromonas* species, and *Erythrobacter litoralis* as the most abundant species, with the rest of the identifications being in much less abundance (Fig 5, S2 File). In sample 1 we identified 204 significantly abundant bacteria, and 229 in sample 2, and 11 differentially abundant when comparing the two samples (S3 File).

### Functional profiling

**Fig 6:**
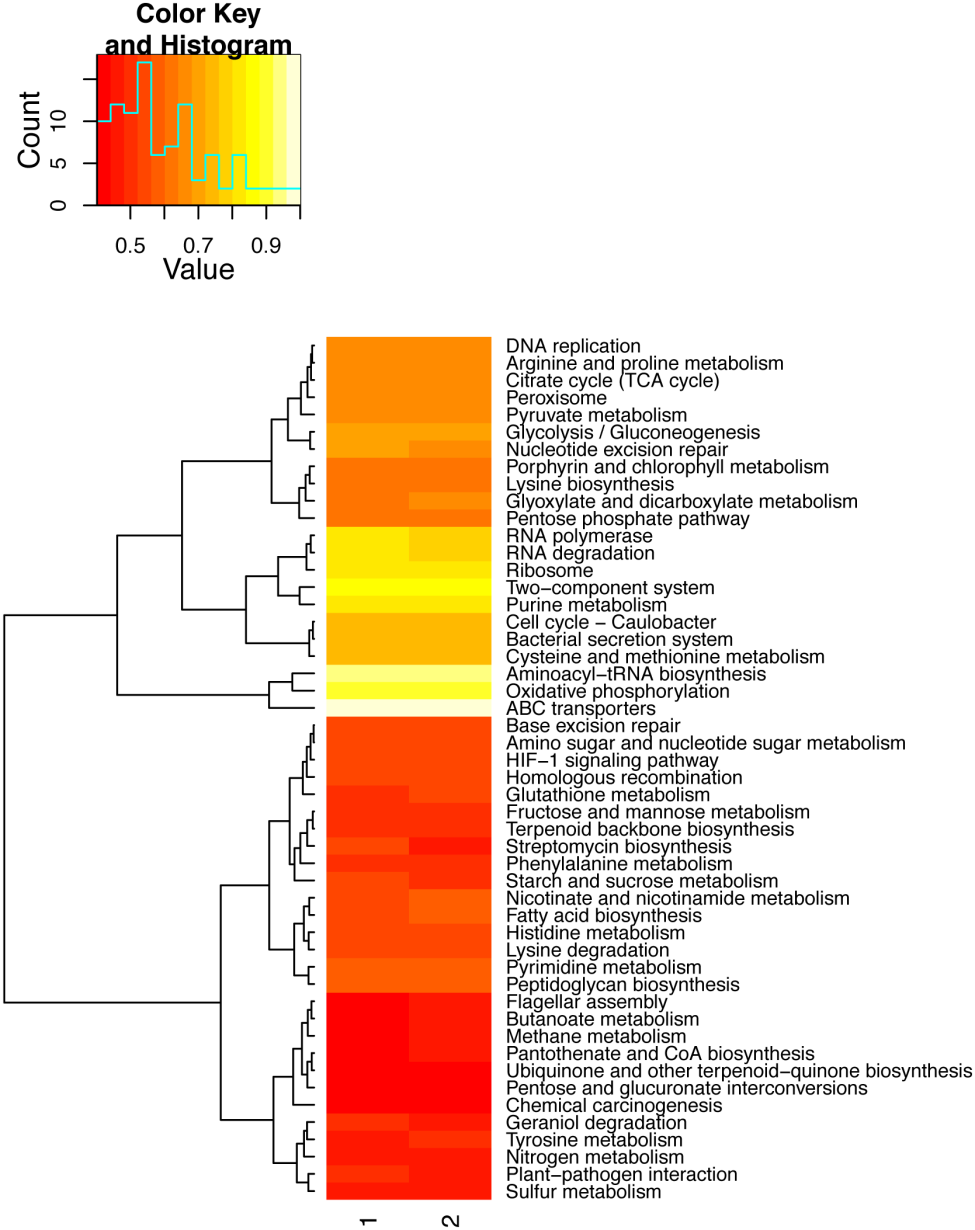
Comparison of the top 50 most abundant functions. Heat map of the top 50 most abundant L3 functions identified with KEGG.

The gene prediction with MG-RAST on both datasets generated a total of 1,392,269 annotated features from sample 1 and 816,342 annotated features from sample 2 (S1 Table). A wide variety of functional categories were identified in both samples, including iron acquisition, sulphur metabolism, and photosynthesis (Fig 6, S4 File, S8A Fig). The genes predicted from the *de novo* assembly are also related to similar functions, as well as nitrogen and carbon fixation, and ureases from uncultured rumen, soil and marine bacteria.

The most abundant functions are the metabolism of amino acids and derivatives, carbohydrates, and clustering-based subsystems—those for which there is evidence that genes belong together but their functions are still unknown. KEGG orthologies (KOs) were also identified (Fig 6B), showing that only very few are related to human diseases. Also, as expected, KOs related to organismal systems (e.g. immune, endocrine, circulatory, and digestive systems) are the least abundant, given that they mostly pertain to multicellular organisms. Also as expected, we found genes related to biofilm formation and heat and cold shock stress response. However, no function was found as statistically significantly abundant within or between the samples.

## Discussion

### DNA binding and leaching on the interior of Tjuv-Ante’s cave speleothem

The DNA adsorption capacity of the opal-A and calcite present in the speleothem could have provided an appropriate DNA binding surface in the environmental conditions of Tjuv-Ante’s cave. In spite of the components of this speleothem, which pose DNA extraction challenges, we were able to generate the present datasets. Although contamination from the outside of the speleothem during the drilling process cannot be completely excluded, this dataset represents one of the first metagenomic studies from the interior part of a speleothem.

It is difficult to differentiate the microbial community of the interior from that of the surface of the speleothem, and it cannot be ruled out that some of the identified microorganisms are due to contamination from communities from the surface of the speleothems, as well as from the cave walls, given the likely occurrence of DNA leaching. A study of archeological dog samples contaminated with human DNA found that authentic aDNA is shorter than the longer retrieved fragments which mostly originate from contamination sources [27]. Thus we investigated the distribution of the length of the reads mapping uniquely to bacteria, fungi and protozoa in order to differentiate allochthonous DNA (i.e. derived from contamination and/or DNA from microorganisms trapped within the speleothem during the growth) from autochthonous DNA (DNA from native microorganisms). We hypothesized that a bimodal distribution would be indicative of DNA derived from both sources being present, with those from longer fragments coming from the allochthonous species that are able to grow on the speleothem, while the short fragments derived from aDNA of degraded microorganisms already present during the growth of the speleothem and/or damaged leaked DNA from the surface above the cave. We found a bimodal distribution of the length of the reads from both samples mapping to the databases of bacteria, fungi and protozoa. The majority of the reads fell in the short length range of the distribution (Fig 6). This suggests that the majority of the DNA of our datasets comes from damaged DNA and also potentially suggests that the diversity of autochthonous bacteria adapted to the speleothem conditions is more restricted [42–44].

### Tjuv-Ante’s cave speleothem as biodiversity archive

The use of molecular studies such as shotgun DNA sequencing could in principle allow the detection of macro-organisms to test for the presence of fauna and vegetation from outside the cave. Importantly, speleothems might act as an archive, not only for climate reconstruction by isotope measurements, but also for historical biodiversity of the area above the cave. Thus we tested our metagenomic datasets for identification of the paleo diversity from above the cave.

We identified land plants, which could come from above the cave, and marine taxa (such as fish, macro algae, mollusks, crustaceans, and corals). The cave is only 20 km away from the coast of the Baltic Sea, and the speleothem was formed after the uplift of the cave, so it has not been in contact with waves. In light of this, we suggest two potential explanations for this result. First, that DNA from marine organisms has been transported to the cave by air dispersion. Second, that these results could be due to misidentification of some taxa.

Given the damage patterns of the long and short reads mapping to bacteria (Fig 3 and S1 Fig), it was not possible to differentiate real aDNA from modern DNA derived from autochtonous microbes living in the speleothem. In aDNA an excess of C-T and G-A substitutions are expected at the extremes of the reads [45,46], however we observed all types of substitutions at similar proportions. Furthermore, the contigs assembled with Ray Meta are suggested to derive mainly from long DNA strands of a modern origin from either endogenous species or derived from recently leaked DNA from above the cave. Thus, reads mapping to these contigs would not be expected to present damage. However, we also observe various types of damage patterns in those reads (S2 and S3 Figs). Also, there are no analyses that can be implemented to unequivocally differentiate endogenous from leached DNA, which is very likely to occur in this substrate. For instance, the various identified worms, spiders, and insects (S6 Fig) might come from natural cave inhabitants, thus representing contamination from the surface of the speleothem.

These observations show that both modern and the possible aDNA present in the sample have suffered damage. One possible cause for the damage might be the action of fungi. Fungi play the destructive role in the constructive-destructive interplay of the speleothem formation by secreting enzymes and acids. These fungal secretions might hamper the potential of speleothems as paleoarchives with the use of shotgun metagenomics. Targeted amplification of barcodes such as ribulose-bisphosphate carboxylase, mitochondrial 16S, 12S, cytochrome b, and control region genes could provide more reliable species level resolution and allow a more direct comparison of the fauna and vegetation identified within the speleothem to the current ones above the cave and those on records from the past in order to conclude the evaluation of the potential of speleothems as paleoarchives.

### Molecular and microscopic analyses comparison

These datasets complement previous microscopic analyses performed from this same cave by Lundberg *et al*. 2013 and Sallstedt *et al*. 2014 [5,9]. These metagenomic datasets provide a wider characterization of the microbiota from this speleothem, with our results indicating a higher diversity than that previously reported. The rarefaction curve (S4 Fig) shows that saturation has not been reached, suggesting that even deeper sequencing would likely increase even more the diversity by detecting the less abundant taxa.

*Actinomycetales* have been morphologically detected by the previous microscopic studies performed on this cave, and we also identified them in our datasets as a major speleothem microbial component (Fig 4). The microscopic analyses suggested that they are the dominant bacteria, however we found that they are actually the second dominating organisms, with *Proteobacteria* in the first place. Chemoautotrophs were suggested to exist in this environment in the two previous studies, although they could not identify them. In the metagenomics datasets we could detect some autotrophs such as *Spirochaetes* (see further discussion below). Furthermore, these previous studies highlighted the absence of photosynthetic bacteria, while our study identified various genes related to this process (see further discussion below).

Furthermore, we could identify various algae that could not be identified with microscopic studies. *Nannochloropsis* was the most abundant alga in sample 1 and is known to be undistinguishable by light and electron microscopy given their morphological indistinctive features [47]. It is also known to occur in fresh and dirty water [48], thus its presence can be associated with the percolated water from the exterior into the speleothem. Sample 2 showed *Aureococcus* as the most abundant alga, this is of relevance given that it is known to cause algal blooms [49]. We also identified diatoms (*Bacillariophyta)*, which can be associated to biofilms [50] and were not identified in the microscopic analyses. Altogether, this comparison highlights the greater identification power provided by metagenomics datasets.

### Autotrophic organisms

The identified genes related to iron and sulfur metabolism indicate the presence of autotrophic organisms. It is most probable that such microorganisms exist in the dolerite, which has reduced forms of iron, sulfur and manganese accessible to chemosynthetic microorganisms. The identification of *Spirochaetes*, which are chemoheterotrophic in nature, also supports this indication. Chemoautotrophs are archaea and bacteria living in environments out of reach of sunlight, such as deep-sea vents, where elements with redox potential, such as Fe, Mn, and S, are the only accessible energy sources. Mafic subterranean rocks have been shown to host microbial communities [51], thus, it is probable that the chemosynthetic microorganisms lived in cracks and pore spaces in the dolerite where Tjuv-Ante’s cave was formed. While alive or upon their death they can be transported downwards by percolating fluids into the cave system and into the speleothem where they may serve as carbon source for heterotrophs in the cave.

One possible explanation to the identification of photosynthetic genes on both samples, pointing to the presence of photoautotrophs, is that they originate from the photoautotrophic *Cyanobacteria* and *Chlorophyta* identified in the samples. Even though the sample was taken from the dark zone of the cave, where photosynthesis is not expected to occur, we also identified *Chlorobi*, light-harvesting green-sulfur photoautotrophic bacteria [52] found at low lights and in deep stratified water columns, which could also be conducting photosynthesis in the biofilm. Furthermore, the bedrock above the cave is thin and the surface is only a few meters above the sampled passage. It is thus likely that small fissures and cracks could facilitate the contact with the surface and provide passage for meteoric water to enter the cave, along with microorganisms from the exterior that may then adapt to survive in the speleothem. Survival of photosynthetic organisms in darkness has been documented for various species [53,54]. The identification of bacterial species residents of microbial mats or hydrothermal settings belonging to the *Chloroflexi* phylum further supports this suggestion, given that *Chloroflexi* is a photoheterotroph that can switch to a non-photosynthetic metabolism [55,56].

Another possible explanation to the identification of photosynthetic genes on both samples is that the presence of the photosynthetic genes derives from entire living or recently dead microorganisms from the surface that were transported to the speleothem, incorporated and preserved in the mineral material. This could be the reason for the identified photosynthetic green algae (*Chlorophyceae*) and the various identified plant species.

In order to deeper explore the existence of photosynthetic organisms in the speleothem, we looked for chloroplast related genes in the *de novo* assembly. We identified plant and bacterial chloroplast related genes in both samples. It is known that carbonate precipitation can be caused by photosynthetic organisms, thus it is likely that the two previous possible explanations are responsible for the presence of photosynthetic genes, with the identified *Cyanobacteria* actively carrying out carbonate mineralization [57], and the plant photosynthetic genes derived from leached DNA from above the cave.

### Taxonomic microbial composition from ancient and modern speleothem formation

As for the paleoarchive interpretation, it is important to also take into account DNA leaching when describing features unique to one of the two samples. Interestingly, *Alkaliphilus metalliredigens* QYMF and *Thermoanaerobacterium thermosaccharolyticum* DSM 571 were identified by uniquely mapping long DNA reads and not by any short read only in sample 1. *Alkaliphilus metalliredigens* QYMF is a metal-reducing alkaliphile species [58], and *Thermoanaerobacterium thermosaccharolyticum* DSM 571 employs a variety of enzymes for the efficient degradation of pullulans, which are polysaccharides that favor the formation of biofilms [1,2].

We identified various classes of fungi, including *Agaricomycetes*, *Blastocladiomycetes*, *Pezizomycetes*, *Saccharomycetes*, and *Schizosaccharomycetes*. Some of these identified fungi correspond to plant pathogens. Particularly, *Melampsora larici-populina* 98AG31 was found only in sample 2. This fungus is the one of the most devastating and widespread pathogen of poplars (deciduous flowering plants native to most of the Northern Hemisphere) [59]. The fact that many of them are plant pathogens supports the suggestion of DNA leaching in the speleothem.

The taxonomic comparison from the MG-RAST results reveals some differences at the phylum taxonomic level (Fig 4, S5 Fig). It is interesting to note that although there is a large difference in abundance between the two samples for the top abundant class (sample 1 has 42.89% and sample 2 has 32.19% of its sequences assigned to *Alphaproteobacteria*), this difference is not statistically significant. The only class with a statistically significant (corrected p value < 0.05) difference in abundance was *Verrucomicrobiae*, which is found in fresh water and soil samples, with sample 1 having 1,447 sequences mapping to it and sample 2 has 85 (S2 File).

It is also interesting to note the presence of *Physarum polycephalum* as the most abundant protoan. It is a light-sensitive slime mold known to inhabit shady cool and moist areas [60], such as decaying leaves and wood, thus the dark-zone area of the speleothem provides a suitable substrate for its growth. Notably, *P*. *polycephalum* feeds on fungal spores and bacteria, as well as other microorganisms. This suggests that it might have a role on the speleothem formation believed to occur by seasonal growth by *Actinomycetales* action and degradation by fungi. Importantly, we identified some significantly abundant bacteria (corrected p value < 0.05) that produce antifungal compounds, such as *Streptomyces rapamycinicus* [61], and *Streptomyces violaceusniger* [62].

Among the bacteria found as having a significantly different abundance within the samples (corrected p value < 0.05), we identified various marine bacteria, as well as bacteria isolated from calcareous stones (*Blastococcus saxobsidens* [63]) or rock varnish in deserts (*Geodermatophilus obscurus* [64]), even bacteria able to grow on living fungal hyphae (*Collimonas fungivoras* [65]). We also identified the presence of the biofilm-forming bacteria *Maricaulis maris*, *Stigmatella aurantiaca*, and *Parvibaculum lavamentivorans* [66–68].

### Functional microbial composition from ancient and modern speleothem formation

The level 2 and 3 KEGG and Subsystems hierarchical functional annotations do not reveal any significant differences between or within the two samples (S8A Fig, S4 File). However, sample 1 is richer in KO functions related to metabolism and genetic and environmental information processing (S8B Fig). Given that sample 1 represents a relatively older formation, these results suggests that there might be living microorganisms adapted to live in the relatively more stable conditions of the speleothem area that is not subject to periodic episodes of growth due to bacterial activity and degradation due to fungal activity. The speleothem growth episodes involve extensive formation of biofilms, which are mostly formed by exopolysaccharides and water [69]. Thus, it is interesting to note that we identified functions related to bacterial biofilm formation and exopolysaccharide production (S4 File), such as xanthan, an industrially relevant exopolysaccharide produced by *Xanthomonas campestris* [70], which we also identified in the taxonomic profiling.

The identification of genes related to fluorobenzoate degradation and in general to xenobiotic metabolism on the newer formation part of the speleothem (sample 2) suggests an active competition between destructive agents and other bacteria. Since biodegradation of fluorinated hydrocarbons (which are herbicides, fungicides, and pharmaceuticals) has been poorly studied [71,72], we suggest that future research on this kind of environments would make important contributions to the understanding of such microbial functions. Interestingly, the identification of cold and heat shock stress response suggests the adaptation to the large microclimate temperature change that ranges from -10 to 15°C in Tjuv-Ante’s cave.

Notably, the alpha diversity in sample 1 is lower than in sample 2 (381.111 and 533.045, respectively). This could be due to two reasons. First, DNA from allochthonous species from the surface of the cave could have leached to the speleothem, thus artificially inflating the alpha diversity. Second, there could be fewer bacteria adapted to the low nutrients conditions of the older formation part of the speleothem, since they would require more specialized functions in order to thrive, while the newer formation part of the speleothem contains more nutrients carried by the percolating water, allowing more species to survive. We suggest that both scenarios are possible. Although we identified some specialized bacteria in sample 2 (such as Desulfuromonadales, Chloroflexales, and Prochlorales), other phyla found in harsh environmental settings such as Aquificales, Methanosarcinales, and Halobacteriales were found in higher abundance in sample 1 than in sample 2, thus supporting the first explanation. And the second explanation is supported by the fact that species found mostly in sample 2 include more macro-species (plants and mammals), which likely derive form leached DNA.

### Biomineralization

As discussed above, we identified taxa and functions from each of the types of bacteria known to precipitate carbonate: a) photosynthetic organisms, such as algae and *Cyanobacteria*, b) sulphate reducing bacteria, c) organic acids utilizers, d) microbes involved in the nitrogen cycle (amino acids ammonification, nitrate reduction and hydrolysis or urea) [73], with the calcium precipitation induced by urea hydrolysis being the simplest and most studied method [74,75].

Specifically, we identified some of the most important bacteria capable of biomineralization of calcium carbonate that have also been applied to the industry. Their applications range from the CO_2_ sequestration as a new means to reducing atmospheric CO_2_, to the rescue of buildings of historical value. For example, we identified *Bacillus cereus*, which has been used for improving the compressive strength of cement mortar [76], and *Myxococcus xanthus*, which has been applied to restore limestone buildings [77].

Among the fungi we identified *Fusarium* species. This fungus causes serious damage on historical buildings [78], and bacteria also identified in this study such as *Desulfovibrio desulfuricans*, *D*. *vulgaris*, and *Shewanella oneidensis* have been shown to aid in their restoration by removing the black sulphate crust, by generating a protective calcium oxalate patina on the stone surface, and by inhibiting the rate of calcite dissolution [79–82]. In the genes identified from the *de novo* assembly we could also identify bacterial carbonic hydratase genes. This ubiquitous enzyme is fundamental to processes such as photosynthesis, respiration, ion and CO_2_ transport [83]. Importantly, it has been shown that this enzyme accelerates CO_2_ hydration and thus calcium carbonate precipitation [84]. Thus, the study of environments where microbes are adapted to naturally induce calcium carbonate precipitation, such as the speleothem examined in this study, is of importance and should be further explored.

## Conclusions

Given the provenance of the samples, a cave formed by the action of seawater on igneous rock with calcite as a major component and with traces of opal-A, the datasets presented here are different from other speleothems-derived datasets that have been studied so far. Thus, they are a new source of information for future comparisons to other environments with similar characteristics. Our metagenomic datasets generated from shotgun DNA sequencing from two samples drilled from different vertical locations from the interior of a speleothem in Tjuv-Ante’s cave represent areas of early and relatively recent speleothem formation. The taxonomic identification results agree with previous microscopy reports of a dominance of *Actinobacteria* and fungi, although we discovered a larger biodiversity. Furthermore, we identified variations in the bacterial taxonomic composition between the two different examined speleothem formation periods. Due to the possibility of DNA leaching from above the cave as well as fungi-induced DNA damage, the use of speleothems as biological paleoarchives could not be unequivocally verified without the use of targeted sequencing. Notably, we detected genes related to photosynthesis, iron and sulfur metabolism, suggesting the presence of autotrophic bacteria, as well as microbes known to cause calcium carbonate precipitation. Microbes that cause mineralization have promising potential in a variety of technological applications, thus environments such as the one presented here should be further explored.

## Supporting Information

S1 Fig Damage pattern of the reads from sample 2 mapping uniquely to the bacterial database in MGmapper. A) Damage pattern of all the reads. B) Damage pattern of the subsampled long reads. C) Damage pattern of the subsampled short reads. C to T damage is depicted in red color, and G to A is depicted in blue color. Grey lines represent other nucleotides derived from other types of DNA damage. The orange line represents soft-clipped bases, those that are not aligned to the reference.
(PDF)

S2 Fig Damage pattern of the reads from sample 1 mapping to the *de novo* assembled contigs. A) Damage pattern of the subsampled long reads. B) Damage pattern of the subsampled short reads. C to T damage is depicted in red color, and G to A is depicted in blue color. Grey lines represent other nucleotides derived from other types of DNA damage.
(PDF)

S3 Fig Damage pattern of the reads from sample 2 mapping to the *de novo* assembled contigs. A) Damage pattern of the subsampled long reads. B) Damage pattern of the subsampled short reads. C to T damage is depicted in red color, and G to A is depicted in blue color. Grey lines represent other nucleotides derived from other types of DNA damage.
(PDF)

S4 Fig Rarefaction curves. The red line is from sample 1 and the blue line is from the sample taken from sample 2.
(TIF)

S5 Fig Phylum taxonomic level heat map. The metagenome from sample 1 has the MG-RAST id 4571211.3, and the 4571212.3 id is from sample 2, mapping against the M5NR database. The most abundant phyla are *Bacteroidetes*, *Proteobacteria*, and *Actinobacteria*, while most of the unexpected biodiversity not previously identified by microscopic analyses is present in lower amounts. The abundances are very similar between the two compared samples.
(TIF)

S6 Fig Tree taxonomic comparison. Leaf abundance weights are displayed as stacked bar charts. The maximum taxonomical level is order and the leaves are colored by phylum.
(TIF)

S7 Fig Rank abundance plots. Abundance from the top 20 most abundant phyla. The y-axis plots the abundances of annotations in each phylum on a log scale. A) Metagenome from sample 1 B) Metagenome from sample 2.
(TIF)

S8 Fig Functional profiling. The metagenome from sample 1 has the MG-RAST id 4571211.3, and the metagenome from sample 2 has the id 4571212.3. A) Subsystems hierarchical functional classification of both datasets. B) KO predicted functions.
(TIF)

S1 File Tjuv-Ante’s Cave supplemental information. (DOCX)

S2 File Taxonomic profiling. Excel file containing identified taxa at class and species level. Sheet one contains the counts comparison of the samples at the phylum and class levels (“MG-RAST-TaxonomyPhylumAndClass”). Sheet two (“KEGG-TaxonomyClass”) contains the classes identified with the KEGG database with normalized values, and sheet three (RayMeta-MEGAN) contains the species identified with MEGAN using the predicted genes from the *de novo* assembly.
(XLSX)

S3 File Taxonomic abundance comparison. Excel file containing the significantly abundant bacteria within the two samples (sheet 1 and 2), and the differentially abundant bacteria between the two samples (sheet 3).
(XLSX)

S4 File Functional profiling. Excel file containing the level 2 and 3 functional identifications with KEGG (sheets 1 and 2), and levels 2 and 3 using Subsystems (sheets 3 and 4).
(XLSX)

S1 Table Data cleaning and processing statistics. (DOCX)

S2 Table Taxonomic profiling statistics. (DOCX)

S3 Table MGmapper mapping statistics. (DOCX)
