# Supplementary figures and images for: Metagenomic Analysis from the Interior of a Speleothem in Tjuv-Ante's Cave, Northern Sweden
Source: PLoS One. 2016 Mar 17;11(3):e0151577. doi: 10.1371/journal.pone.0151577 (PMC4795671; doi:10.1371/journal.pone.0151577)

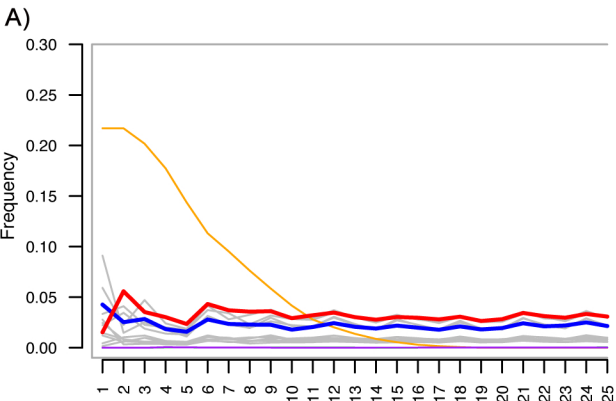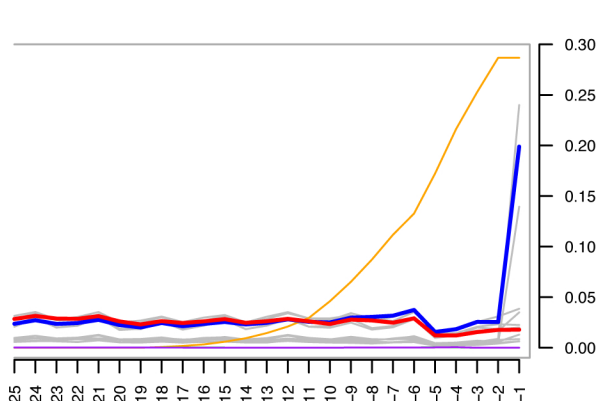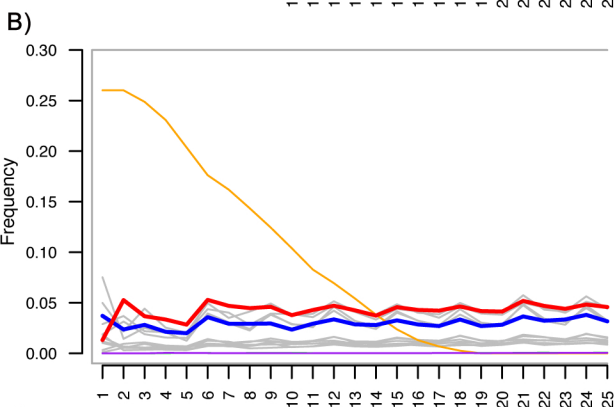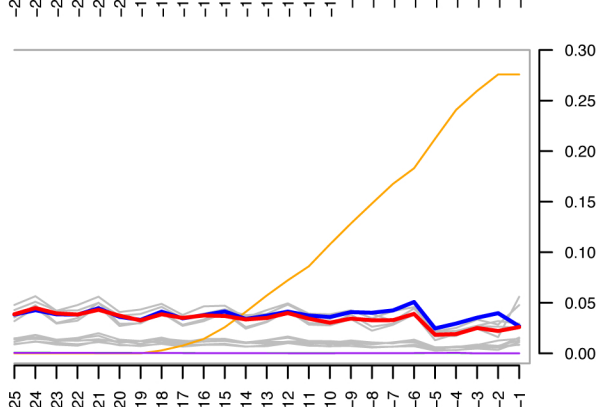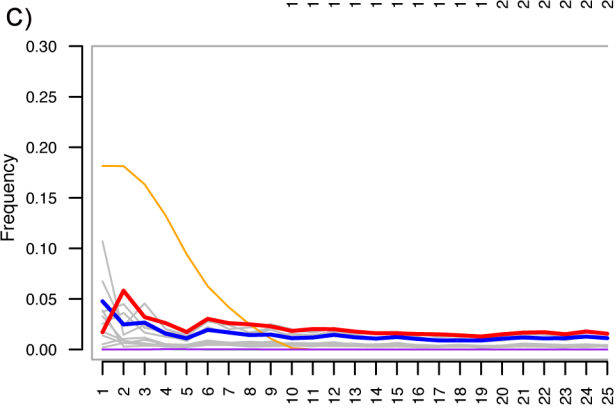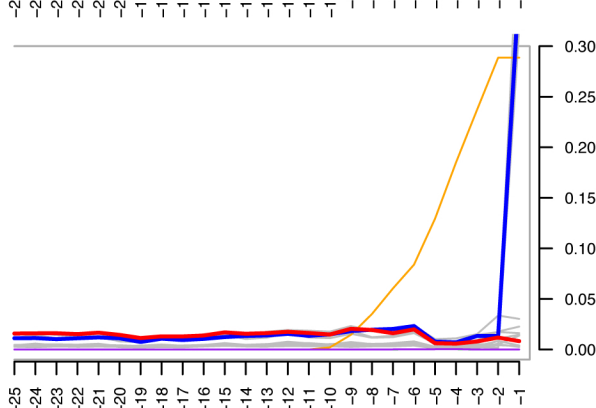

Supplement: S1 Fig — A) Damage pattern of all the reads. B) Damage pattern of the subsampled long reads. C) Damage pattern of the subsampled short reads. C to T damage is depicted in red color, and G to A is depicted in blue color. Grey lines represent other nucleotides derived from other types of DNA damage. The orange line represents soft-clipped bases, those that are not aligned to the reference. (PDF) [file pone.0151577.s001.pdf]

A)

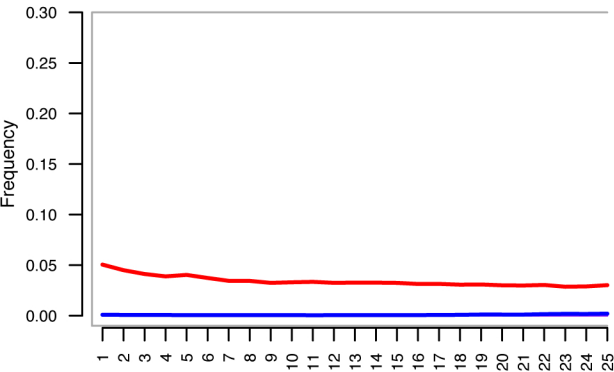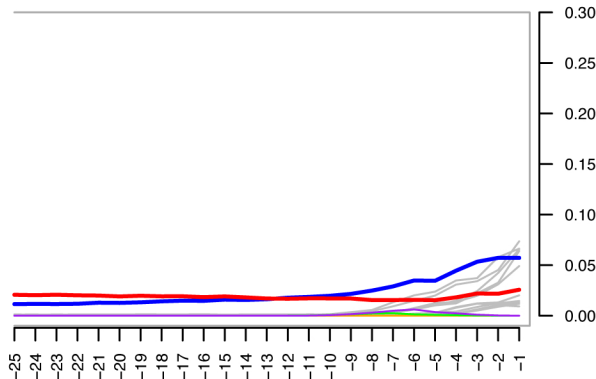

B)

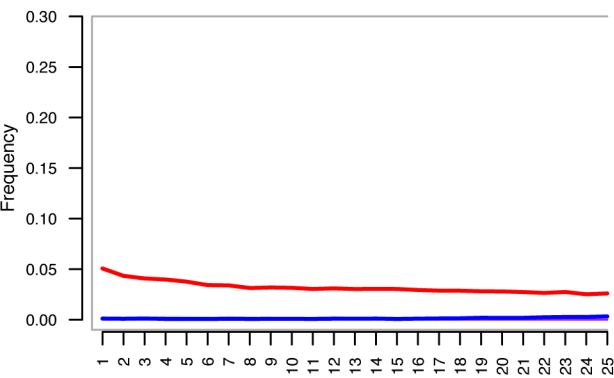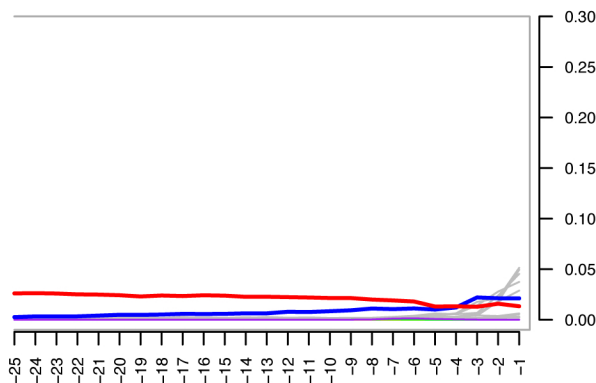

Supplement: S2 Fig — A) Damage pattern of the subsampled long reads. B) Damage pattern of the subsampled short reads. C to T damage is depicted in red color, and G to A is depicted in blue color. Grey lines represent other nucleotides derived from other types of DNA damage. (PDF) [file pone.0151577.s002.pdf]

A)

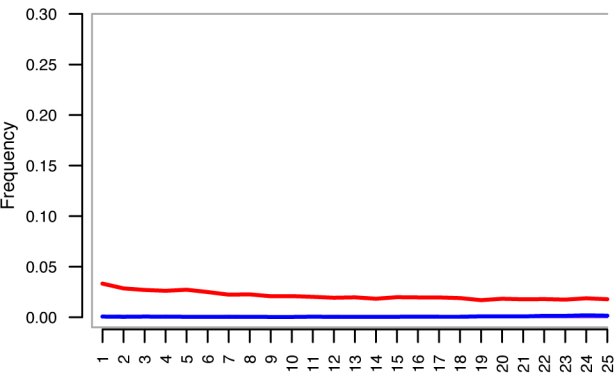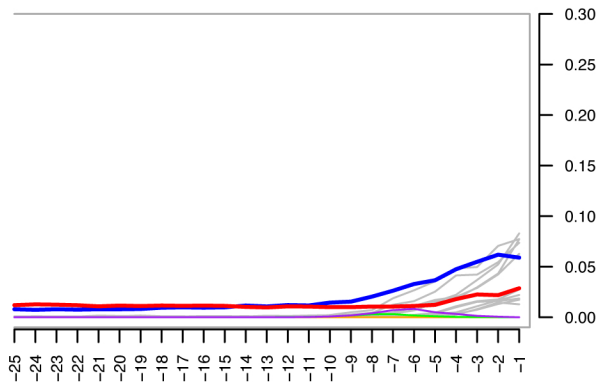

B)

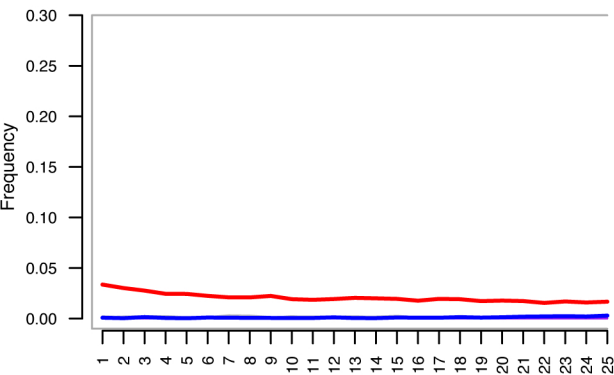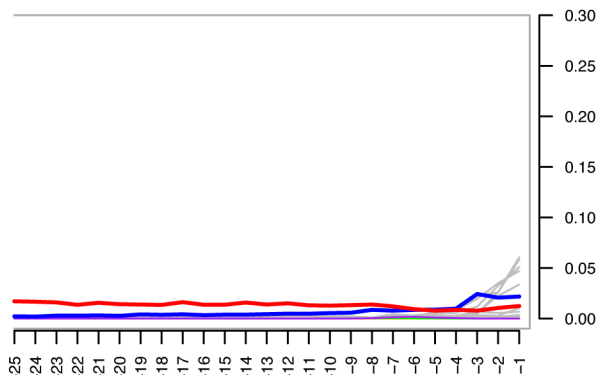

Supplement: S3 Fig — A) Damage pattern of the subsampled long reads. B) Damage pattern of the subsampled short reads. C to T damage is depicted in red color, and G to A is depicted in blue color. Grey lines represent other nucleotides derived from other types of DNA damage. (PDF) [file pone.0151577.s003.pdf]

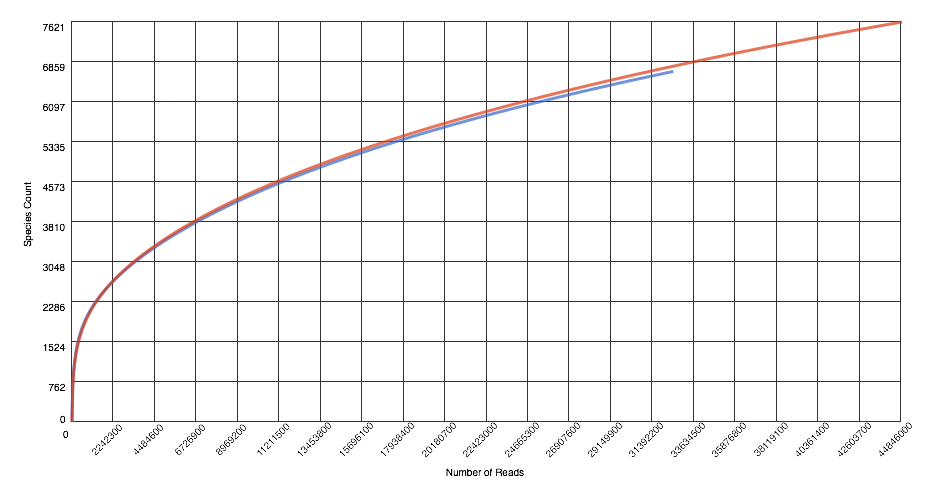

Supplement: S4 Fig — The red line is from sample 1 and the blue line is from the sample taken from sample 2. (TIF) [file pone.0151577.s004.tif]

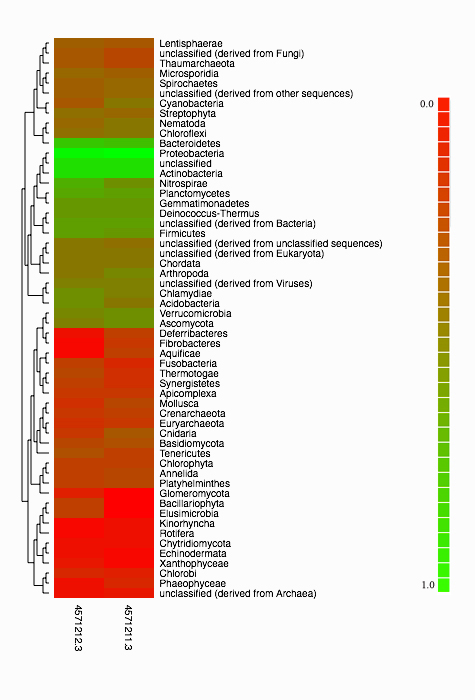

Supplement: S5 Fig — The metagenome from sample 1 has the MG-RAST id 4571211.3, and the 4571212.3 id is from sample 2, mapping against the M5NR database. The most abundant phyla are Bacteroidetes, Proteobacteria, and Actinobacteria, while most of the unexpected biodiversity not previously identified by microscopic analyses is present in lower amounts. The abundances are very similar between the two compared samples. (TIF) [file pone.0151577.s005.tif]

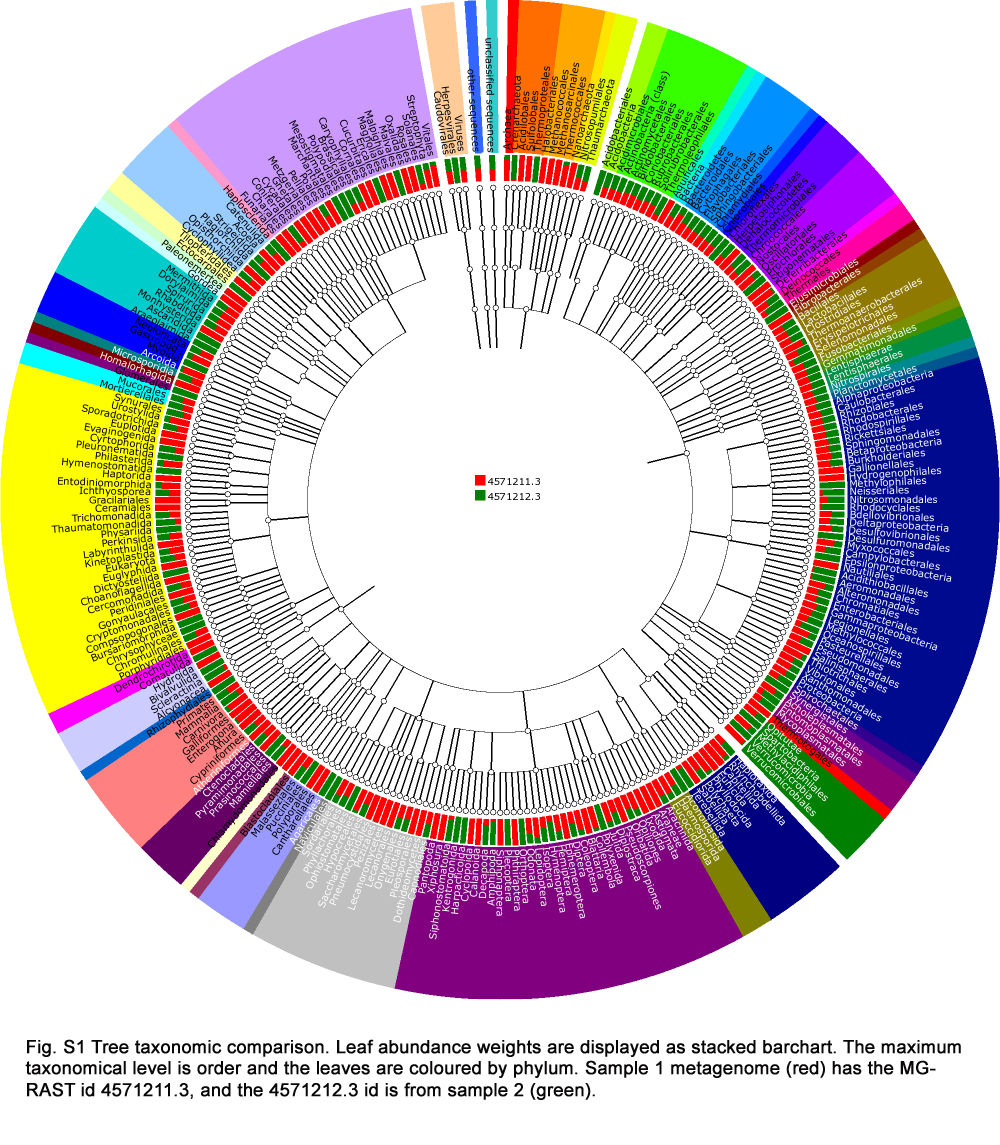

Supplement: S6 Fig — Leaf abundance weights are displayed as stacked bar charts. The maximum taxonomical level is order and the leaves are colored by phylum. (TIF) [file pone.0151577.s006.tif]

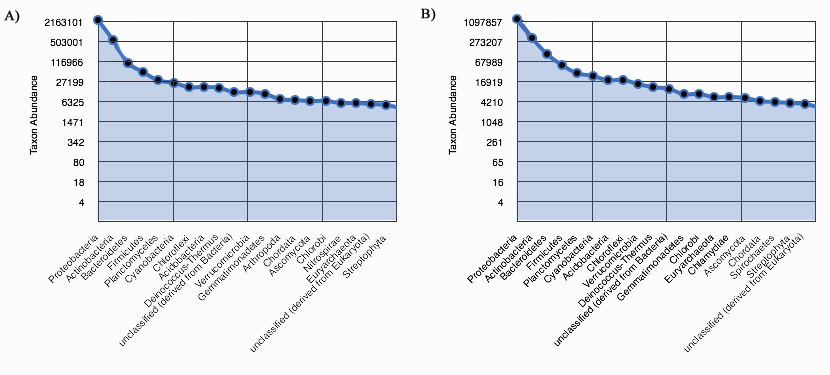

Supplement: S7 Fig — Abundance from the top 20 most abundant phyla. The y-axis plots the abundances of annotations in each phylum on a log scale. A) Metagenome from sample 1 B) Metagenome from sample 2. (TIF) [file pone.0151577.s007.tif]

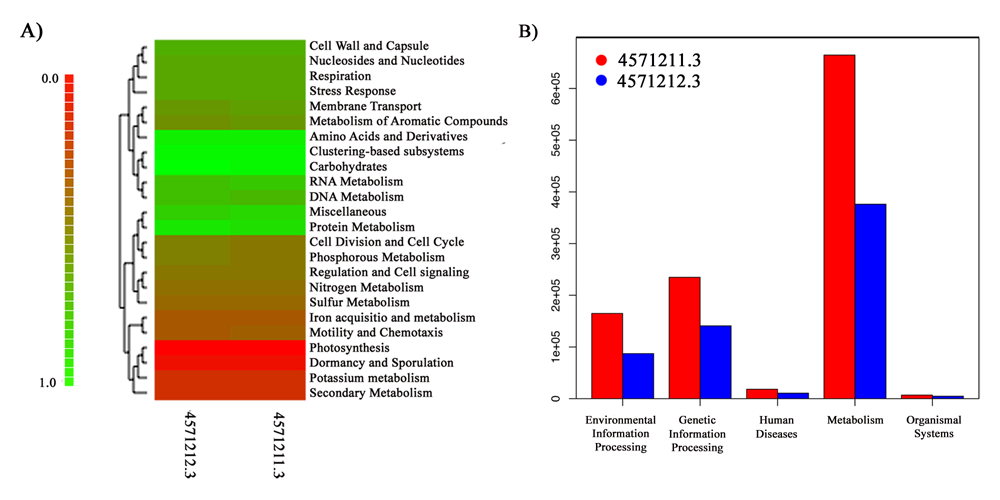

Supplement: S8 Fig — The metagenome from sample 1 has the MG-RAST id 4571211.3, and the metagenome from sample 2 has the id 4571212.3. A) Subsystems hierarchical functional classification of both datasets. B) KO predicted functions. (TIF) [file pone.0151577.s008.tif]
