# Supplementary material for: Metagenomic Analysis from the Interior of a Speleothem in Tjuv-Ante's Cave, Northern Sweden
Source: PLoS One. 2016 Mar 17;11(3):e0151577. doi: 10.1371/journal.pone.0151577 (PMC4795671; doi:10.1371/journal.pone.0151577)
Supplement: S1 File — (DOCX) [file pone.0151577.s009.docx]

**Tjuv-Ante’s Cave supplemental information**

Tjuv-Ante's cave is a small sea abraded cave situated in Storrisberget's Nature Reserve (N 63° 35.6', E 19° 22.8', altitude 90m above sea level), and has been described and surveyed by Sjöberg (1982). The cave is about 30m long, with a width of about 0.5 to 1.5m. It is formed along a dolerite dyke in granite gneiss by the action of high-energy waves. The formation was initiated when the sea level reached the uppermost part of the dyke due to the isostatic uplilft following the Weichselian glaciation about 8000 years before present (BP); and ending about 6,500 years BP when the crevice with the cave was out of reach for the wave action (Sjöberg, 1982).

The speleothems were sampled from the dark zone in the inner part of Tjuv-Ante's Cave. The sampling methodology has been described in Sallstedt et al. (2014). All speleothems in the cave were precipitated on the dolerite, either on the ceiling or the wall, in the form of crusts or cylindrical speleothems (popcorn-like or coral-like; Sallstedt et al., 2014). A modern biofilm, not directly associated with the speleothems but found on the dolerite in close proximity (less than 2m away) on the dolerite was also collected. In the cave it forms bright white colonies, somewhat reminiscent of lichens, but dominated by filamentous microorganisms morphologically corresponding to Actinobacteria (Sallstedt et al., 2014).

The speleothems were studied using Environmental Scanning Electron Microscopy (ESEM) coupled with Energy Dispersive Spectrometry (EDS) as well as by Scanning Electron Microscopy (SEM) as described in Sallstedt et al. (2014). The microscopy revealed a growth-ring like zonation of the interior of the speleothem, with alternating dark and light laminae. The EDS analysis indicated that the lighter laminations are almost pure calcium carbonate (calcite), while the darker laminations have high silica (c. 20 weight%), magnesium (7-15 weight%) and carbon contents (Sallstedt et al., 2014). δ13C measurments were taken from two speleothems (Sallstedt et al., 2014), with the δ13C in the range between 9.0 and 11.0 indicating an inorganic source of the carbon (Sharp, 2007). A sample of the speleothem was ^14^C-dated to 1259±30 years BP (Sallstedt et al., 2014). The ESEM and the SEM also revealed a surface covering biota consisting of a variety of filamentous microorganisms spanning a large size range. The thinnest filaments (less than 1μm in diameter) morphologically correspond with Actinobacteria, while the larger filaments (about 10μm in diameter) are similar to filamentous fungi (Sallstedt et al., 2014). Groups of coccoids were present on the surface of the speleothem, also consistent with Actinobacteria (Sallstedt et al., 2014).

**References**

Sallstedt, T., Ivarsson, M., Lundberg, J., Sjöberg, R., Vidal Romaní, J.R. 2014. Speleothem and biofilm formation in a granite/dolerite cave, Northern Sweden. International Journal of Speleology 43: 305-313. <http://dx.doi.org/10.5038/1827-806X.43.3.7>

Sharp, Z. 2007. Principles of stable isotope geochemistry. Pearson Education, Upper Saddle River.

Sjöberg, R. 1982. Tunnelgrottor i södra Västerbotten: morgologiska och morfogenetiska studier. Gerum Geografiska Rapporter A:31. 93 p.
