# Supplementary material for: Metagenomic Analysis from the Interior of a Speleothem in Tjuv-Ante's Cave, Northern Sweden
Source: PLoS One. 2016 Mar 17;11(3):e0151577. doi: 10.1371/journal.pone.0151577 (PMC4795671; doi:10.1371/journal.pone.0151577)
Supplement: S1 Table — (DOCX) [file pone.0151577.s013.docx]

**S1 Table. Data cleaning and processing statistics with MG-RAST.**

|  | Sample 1 | Sample 2 |
| --- | --- | --- |
| Initial number of sequences | 44,846,754 | 32,491,609 |
| Replicated sequences | 9,819,641 (21.9%) | 5,966,639 (18.4%) |
| Average read length | 93 | 93 |
| Sequences passing the QC | 30,005,882 (66.9%) | 23,375,177 (71.9%) |
| Predicted protein features with annotation | 1,708,668 | 1,026,122 |
| Predicted protein features with functional categories | 1,392,269 features (81.5% of annotated features) | 816,342 features (79.6% of annotated features) |
| Predicted protein features without annotations | 23,558,081 (93.2% of features) | 18,569,282 (94.8% of features) |
