# Supplementary material for: Metagenomic Analysis from the Interior of a Speleothem in Tjuv-Ante's Cave, Northern Sweden
Source: PLoS One. 2016 Mar 17;11(3):e0151577. doi: 10.1371/journal.pone.0151577 (PMC4795671; doi:10.1371/journal.pone.0151577)
Supplement: S2 Table — (DOCX) [file pone.0151577.s014.docx]

**S2 Table. Taxonomic profiling with MG-RAST statistics.**

|  | Sample 1 | Sample 2 |
| --- | --- | --- |
| Sequences pre-screened with 70% identity | 9,771,866 | 6,845,168 |
| *De novo* clusters with 97% identity | 462506 | 462506 |
| Number of BLAT queried sequences with a hit | 22543 | 22543 |
| Number of BLAT hits | 33457 | 28667 |
