# Supplementary material for: Metagenomic Analysis from the Interior of a Speleothem in Tjuv-Ante's Cave, Northern Sweden
Source: PLoS One. 2016 Mar 17;11(3):e0151577. doi: 10.1371/journal.pone.0151577 (PMC4795671; doi:10.1371/journal.pone.0151577)
Supplement: S3 Table — (DOCX) [file pone.0151577.s015.docx]

| Mapping mode | Database | % reads | | No Reads | | Reads available | |
| --- | --- | --- | --- | --- | --- | --- | --- |
|  |  | Sample 1 | Sample 2 | Sample 1 | Sample 2 | Sample 1 | Sample 2 |
| Fullmode | notPhiX | 100 | 100 | 37724025 | 28027864 | 37726455 | 28029078 |
| Chainmode | Human | 0.046 | 0.034 | 17407 | 9436 | 37724025 | 28027864 |
| Chainmode | Bacteria | 2.675 | 2.102 | 1008956 | 589148 | 37698920 | 28015322 |
| Chainmode | Virus | 0.001 | 0.001 | 249 | 180 | 36321327 | 27193495 |
| Chainmode | Fungi | 0.019 | 0.014 | 7108 | 3823 | 36320933 | 27193197 |
| Chainmode | Protozoa | 0.008 | 0.007 | 2908 | 1986 | 36312440 | 27188675 |
| Chainmode | Unmapped | 97.25 | 97.84 | 36687397 | 27423291 |  |  |

**S3 Table. MGmapper mapping statistics**
